# Diarrhea in Under Five Year-old Children in Nepal: A Spatiotemporal Analysis Based on Demographic and Health Survey Data

**DOI:** 10.3390/ijerph17062140

**Published:** 2020-03-23

**Authors:** Ruixue Li, Yingsi Lai, Chenyang Feng, Rubee Dev, Yijing Wang, Yuantao Hao

**Affiliations:** 1 School of Public Health, Sun Yat-sen University, Guangzhou 510080, China; lirx6@mail2.sysu.edu.cn (R.L.); laiys3@mail.sysu.edu.cn (Y.L.); wangyj77@mail2.sysu.edu.cn (Y.W.); 2 Sun Yat-sen Global Health Institute, Sun Yat-sen University, Guangzhou 510275, China; Rubee@mail.sysu.edu.cn

**Keywords:** diarrhea, spatiotemporal analysis, child, influencing factor

## Abstract

Background: Diarrhea in children under five years of age remains a challenge in reducing child mortality in Nepal. Understanding the spatiotemporal patterns and influencing factors of the disease is important for control and intervention. Methods: Data regarding diarrhea prevalence and its potential influencing factors were extracted from the Demographic and Health Surveys in Nepal and other open-access databases. A Bayesian logistic regression model with district-specific spatio-temporal random effects was applied to explore the space and time patterns of diarrhea risk, as well as the relationships between the risk and the potential influencing factors. Results: Both the observed prevalence and the estimated spatiotemporal effects show a decreasing diarrhea risk trend from 2006 to 2016 in most districts of Nepal, with a few exceptions, such as Achham and Rasuwa. The disease risk decreased with mothers’ years of education (*OR* 0.93, 95% Bayesian Credible Interval (BCI) 0.87, 0.997). Compared to spring, autumn and winter had lower risks of diarrhea. The risk firstly increased and then decreased with age and children under 12–24 months old were the highest risk group (*OR* 1.20, 95% BCI 1.04, 1.38). Boys had higher risk than girls (*OR* 1.24, 95% BCI 1.13, 1.39). Even though improved sanitation wasn’t found significant within a 95% BCI, there was 93.2% of chance of it being a protective factor. There were no obvious spatiotemporal clusters among districts and each district tended to have its own spatiotemporal diarrhea prevalence pattern. Conclusions: The important risk factors identified by our Bayesian spatial-temporal modeling provide insights for control and intervention on children diarrhea in Nepal. Special attention should be paid to high risk groups of children and high risk seasons, as well as districts with high risk or increased trend of risk. Effective actions should be implemented to improve sanitation and women’s education level. District-specific control planning is recommended for local governments for effective control of children diarrhea in Nepal.

## 1. Introduction

Addressing inequities and disparities in child health is necessary to protect children’s right to survive [1]. Goals are set to reduced child mortality in both the Millennium Development Goals and the subsequent Sustainable Development Goals by the United Nations [2,3]. Diarrhea, one of the leading causes of death in children, is a major threat to child health [4]. Childhood diarrhea is defined as the passage of three or more loose or watery stools per 24 h or an increase in stool frequency or liquidity considered abnormal by the mother [5]. In 2016, more than 5.6 million children under five years of age died worldwide, of which 8.4% were attributed to diarrhea [6]. Furthermore, it was estimated that globally there were 1.73 billion episodes of diarrhea in children in 2010 and 70 thousand episodes led to death in 2011 [7].

Nepal has made tremendous advances in child health and achieved the corresponding Millennium Development Goal of reducing child mortality by two thirds in 2015 [8]. However, the mortality rate of children under five years old was still high (i.e., 32 deaths per 1000 live births in 2018), which was around five times higher than that in high-income countries such as the United States of America (i.e., 6 deaths per 1000 live births in 2018) [9,10]. Diarrhea remains the leading cause of morbidity and mortality of children under five years old [11]. Besides, it leads to stunting and malnutrition of children [12,13]. The prevalence of diarrhea within two weeks varied geographically across the country, ranging from 3.7% to 9.0% in different provinces according to the recent Nepal Demographic and Health Survey (NDHS) in 2016 [14]. According to the WHO, there were 1193 deaths under five caused by diarrhea in Nepal in 2017 [15]. A survey even showed that more than one third of investigated children had diarrhea within the two weeks preceding the survey in a rural community of southern Nepal [16]. Compared to other South Asias countries (e.g., Bangladesh), the risk of diarrhea in children under five is higher in Nepal [17].

Many factors were identified as influencing factors of diarrhea in children under five years old. Poor household environments, such as unsafe drinking water and poor sanitation, cause a large number of diarrheal cases, contributing to the death of children in low- and middle-income countries [18]. Households with better economic conditions showed lower incidence of childhood diarrhea [19]. Maternal-related factors, such as maternal education levels and breastfeeding, were associated with diarrhea prevalence [20]. In addition, personal behaviors (e.g., personal hygiene habits) also play an essential role in childhood diarrhea [18]. Budhathoki and colleagues did a framework analysis of eco-social and behavioural determinants of children diarrhea in Nepal, eliciting age, gender, hand-washing behaviour, nutritional status of children, education of mothers, water and sanitation, healthcare services, cultural and societal values and income of the household as the identified determinants [21]. Furthermore, seasonality, environment and climatic factors (e.g., temperature, elevation, humidity and flooding) showed important relationships with diarrhea risk in some studies [22,23,24].

Understanding the spatiotemporal patterns and the major influencing factors of diarrhea in under five year-old children in Nepal is important for disease control and health resource allocation, which will further help reduce child health inequities and disparities. A report of the Nepal Demographic Health Survey summarized the diarrhea prevalence in children at the province level [14]. However, it’s necessary to present the diarrhea risk at a smaller administrative level (e.g., district level) to better show the within-province heterogeneity, which is more helpful for spatially-targeted intervention. On the other hand, previous studies mostly used simple or multiple logistic regressions to explore the relationships between diarrhea and potential risk factors in Nepal, which may ignore the effects of geographical heterogeneity [20]. Bayesian hierarchy models introduce random effects to capture the variance due to space, time and unknown or unobserved influencing factors, to thus provide better estimates of the relationships between the disease risk and the known explanatory variables [25]. Such a modeling approach is one of the most rigorous methods to analyse the space and temporal patterns of disease risk and the corresponding influencing factors [26]. A series of models have been applied to understand the spatial and temporal variations of diarrhea in many regions using diarrhea survey data [27,28,29]. However, to our knowledge, there hasn’t been any study exploring the spatiotemporal patterns of diarrheal risk in Nepal. In this study, we aim to examine these patterns and to identify the important influencing factors in Nepal, based on the open-access Demographic Health Survey (DHS) data with application of Bayesian spatial-temporal models.

## 2. Materials and Methods

### 2.1. Study Area

**Figure 1:**
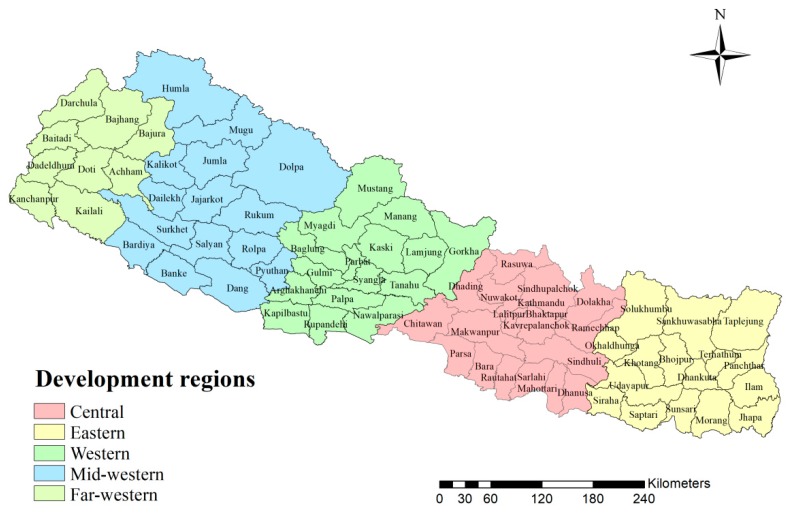
Development regions and districts in Nepal.

Nepal, a low-income country in South Asia [30], nestled in the foothills of the Himalayas. It occupies an area from 26°22′ to 30°27′ north latitude and 80°4′ to 88°12′ east longitude, with elevations ranging from 90 m to 8848 m. Topographically, Nepal is divided into three distinct ecological zones, that is mountain, hill, and terai (i.e., plains). Besides, there are five development regions in Nepal, which are the Eastern, the Central, the Western, the Mid-western and the Far-western development regions. Furthermore, Nepal consists of 75 districts distributed across different ecological zones and development regions. We used the district-level division as the geographical unit summarizing the diarrhea prevalence. Figure 1 shows development regions and 75 districts in Nepal [31].

### 2.2. The Data

The major data in this study was extracted from the public Demographic and Health Surveys (DHS) database [32]. Data used in our study come from public data and the data are anonymized so that no ethical approval was sought. The Institution collected the data are responsible for securing the appropriate ethical approval prior to data collection. NDHS were representative national household surveys conducted every five years in Nepal. The three most recent data from the survey year 2006, 2011 and 2016 were applied. There was also a survey undertaken in 2001. However, data of many corresponding potential risk factors that we planned to explore was missing in the survey, thus the disease data in 2001 was not included in this study. NDHS used two- (in 2006 and 2011) or three-stage (in 2016) sampling method to extract representative samples, which were detailed in the corresponding NDHS reports [14,33,34]. Over 100 peer-review articles concerning on various health issues were published, based on DHS data. We belied the survey data is reliable, as quality control measures were undertaken during the whole process of investigation: before the field interview, pretest was carried out and the field workers were well-trained; quality controllers were assigned to monitor the fieldwork during survey; and as soon as data collection was completed, the data files were registered and checked for inconsistencies, incompleteness, and outliers [34].

Information on basic demographic and health topics was collected through questionnaires in the NDHS. All NDHS questionnaires can be obtained through NDHS reports. The major variables in this study were derived from the NDHS Women’s Questionnaire, which was used to collect information from women aged 15–49 years old on topics including antenatal, delivery, postnatal care, immunization and the childhood illnesses of their children. Children were referred to have diarrhea within two weeks if the mothers answered “yes” to the question “if the child having symptoms of diarrhea in two weeks preceding the survey”. This indicator is the major indicator in the current study and treated as dependent variable in the subsequent modeling analysis.

According to the framework on eco-social and behavioural determinants of children diarrhea proposed by Budhathoki and colleagues, as well as other influencing factors identified by other studies through a literature review [16,21,35], we considered a range of factors, data of which can be either obtained from NDHS or other open-access databases, as potential risk factors in this study. These factors included children’s personal factors (i.e., child’s age, child’s gender and whether the child living with mother), maternal-related factors (i.e., mother’s years of education, mother’s age at birth of the child and number of children under five years old in the family), socio-economic factor (i.e., wealth index, type of place of residence, residing status), and household-related factors (i.e., water source, sanitation facility and fuel type), information of which obtained from the NDHS Women’s questionnaire. The water source, sanitation facility and fuel type were classified to two categories (i.e., improved and unimproved for water source and sanitation facility, and solid and nonsolid for fuel type) according the DHS’s definitions [14,33,34]. According to the seasonal characteristics of Nepal [36], we classified the survey season as spring (February to April), summer (May to July), autumn (August to October) and winter (November to January), based on the month of the survey.

Environmental and climatic data including elevation, normalized difference vegetation index (NDVI), moisture, water bodies and land surface temperature (LST) in the daytime and at night were obtained from readily open-access data sources (Table 1). The NDVI and LST data were averaged for each year. The NDVI, LST and elevation data were aligned over a grid of 5 × 5 km spatial resolution. Data at the survey clusters were extracted and assigned to the corresponding investigated individuals. Distances to the nearest fresh water bodies were calculated as absolute distances according to coordinates of survey clusters and positions of water bodies. The above data processing procedures were done using ‘raster’ package in R version 3.5.0 (Robert J. Hijmans, CA, USA).

### 2.3. Statistical Analysis

Single-factor analysis was conducted to compare the differences of potential influencing factors between children with diarrhea and those without. Chi-square tests were used for categorical variables, while *t* tests or Wilcoxon rank-sum tests were applied for continuous variables, depending on the normality of the variables: if the normality test showed the continuous variables follow a normal distribution, a *t* test was used, otherwise a Wilcoxon rank-sum test was applied. The significance level was set to 0.05. Factors with *p*-values larger than 0.1 were put forward to the following Bayesian spatial-temporal modeling.

Moran’s ***I*** was used to detect the overall spatial autocorrelation of diarrhea risk for each survey year in children under five years old between districts using the formula $I_{t}=\frac{N_{t}}{S}\frac{\sum_{i=1}^{n}\sum_{j=1}^{n}\omega_{ij}\left(y_{it}-{\bar{y}}_{t}\right)\left(y_{jt}-\bar{y_{t}}\right)}{\sum_{i=1}^{n}{\left(y_{it}-\bar{{\bar{y}}_{t}}\right)}^{2}}$, where $S=\overset{n}{\underset{i=1}{\sum }}\overset{n}{\underset{j=1}{\sum }}\omega_{ij}$ [37]. Here $y_{it}$ and $y_{jt}$ are the observed prevalence of diarrhea among children under five years old in the *i*^th^ and *j*^th^ districts in survey year t (t = 2006, 2011 or 2016), respectively. $N_{t}$ is the total number of the districts, and $\omega_{ij}$ the spatial neighborhood weight for the district *i* and *j*. If *i* and *j* are adjacent neighbors, $\omega_{ij}=1$, otherwise $\omega_{ij}=0$. Significance of the coefficients was assessed by calculating the *z*-score and the corresponding *p*-value [38]. ArcGIS 10.2 (Esri, CA, USA) was used to conduct the analysis.

In the next step, the Bayesian logistic regression model with district-specific spatial-temporal random effects was applied to explore the space and time patterns of diarrhea risk, as well as the relationships between the risk and the potential influencing factors. For the *i*^th^ individual belonging to the *j*^th^ district in the time *t* (*t* = 1, 2 or 3 representing the survey year 2006, 2011 or 2016, respectively), we assumed the disease status $Y_{ijt}$ ($Y_{ijt}=1$ indicating with diarrhea and $Y_{ijt}=0$ without diarrhea) arises from a Bernoulli distribution, that is $Y_{ijt}∼\mathrm{Bern}\left(p_{ijt}\right)$. In particular, $\mathrm{logit}\left(p_{ijt}\right)=\alpha +X_{ijt}^{T}\beta +\delta_{jt}$, where α, $X_{ijt}$ and β were the intercept, the vector of covariates and the vector of coefficients. $\delta_{jt}$ was the district-specific spatial-temporal random effect in district *j* of survey time *t*, representing the additional risk due to unknown or unobtainable factors after accounting for the risk factors we considered in the model. We assumed δ follows a zero-mean Gaussian distribution with a precision matrix $R/\sigma^{2}$, Here $\sigma^{2}$ indicates the variance and $R=R_{t}⊗R_{d}$ was assumed to factorized as the Kronecker product of the matrices corresponding to the temporal and the spatial effects which interact [39].

There are four types of interaction: type I assumes unstructured effects for both space and time with $R_{t}=I$ and $R_{d}=I$ (I the unstructured unit matrix), thus they interact in the form as $\delta_{jt}∼N\left(0,1/\sigma^{2}\right)$, assuming independent effects for each district in each survey time; type II combines the structured temporal main effect and the unstructured spatial effect with $R_{d}=I$ and $R_{t}$ a structure form such as autoregressive of order 1 (AR1), assuming independent effects across districts but relative effects of time for each district; type III combines the unstructured temporal effect and the spatially structured main effect with $R_{t}=I$ and $R_{d}$ a structure form such as the intrinsic conditional autoregressive (iCAR), assuming relative effects of districts in each survey time but independent effects across survey time; and type IV combines both structured spatial and temporal effects, assuming relative effects across districts and time. If the Moran’s ***I*** coefficients suggest autocorrelation of diarrhea risk between districts, the spatial matrix $R_{d}$ was assigned with a neighboring structure defined through the iCAR specification [40], otherwise I was assigned, indicating exchangeable random effects. We considered two possible structures for the temporal matrix $R_{t}$, that is a structure of AR1 with coefficient ρ under the assumption that diarrhea risk of each district correlated on time, and a unit matrix I indicating independent of diarrhea risk between survey years. If the fitting result shows ρ significant, the AR1 structure was adopted for the final model, otherwise, the I was used.

Bayesian inferential framework was adopted to estimate the parameters as well as hyperparameters. Minimally informative priors were set as following: $\alpha ,\beta_{k}∼N\left(0,\;1000\right)$, $\log\left(\frac{1}{\sigma^{2}}\right)∼gamma\left(1,\;0.01\right)$ and log((1+ρ)/(1−ρ))∼N(0,0.15). Model fitting was undertaken through the integrated nested Laplace approximation (INLA) method [41] using the R-INLA package (available at www.r-inla.org) with R version 3.5.3., (R Core Team, Vienna, Austria).

To present the relative spatial-temporal risk of districts, we estimated the *OR* of diarrhea risk for each district compared to the estimated country-level prevalence in the most recent survey year 2016. The methodology is as following: (1) through the individual-level model, the prevalence of individuals with possible combinations of covariates was estimated in each district of each survey year; (2) district-level prevalence were further summarized by weighted averaging the individual-level prevalence, where weights were calculated by multiplying the district-specific proportions of covariates that the combinations referred to; (3) the estimated relative risk of district *j* in survey year *t* was presented as $OR_{jt}$, which calculated as $OR_{jt}=\exp(\mathrm{logit}$(prevalence estimated in district *j* of survey year *t*)−logit(population-weighted country-level prevalence in survey year 2016)). To better show the temporal change of diarrhea risk for districts, the corresponding *OR* were calculated, that is:

$$\begin{array}{c} OR_{j,t_{u-v}}=\exp(\mathrm{logit}\left(\mathrm{prevalence}\;\mathrm{estimated}\;\mathrm{in}\;\mathrm{district}\;j\;\mathrm{of}\;\mathrm{survey}\;\mathrm{year}\;t_{u}\right)-\\ \mathrm{logit}\left(\mathrm{prevalence}\;\mathrm{estimated}\;\mathrm{in}\;\mathrm{district}\;j\;\mathrm{of}\;\mathrm{survey}\;\mathrm{year}\;t_{v}\right)), \end{array}$$

indicating the relative risk for survey year $t_{u}$ compared to that for $t_{v}$ for district j. All the calculations were based on randomly sampling of 500 samples from posterior distributions, thus the median, the standard deviation (*SD*) of OR and the possibility of OR larger than one were obtained and further presented in the map of Nepal.

The variance explained by covariates and that by random effects (δ) of the model was assessed using pseudo $R^{2}$. The temporal changes of $\delta_{jt}$ were calculated as $\exp\left(\delta_{jt_{u}}-\delta_{jt_{v}}\right)$. Separate models by year were run and the same method was used to detect the variance explained by covariates and random effect in each survey year.

## 3. Results

### 3.1. Summary of NDHS on Children Diarrhea

**Figure 2:**
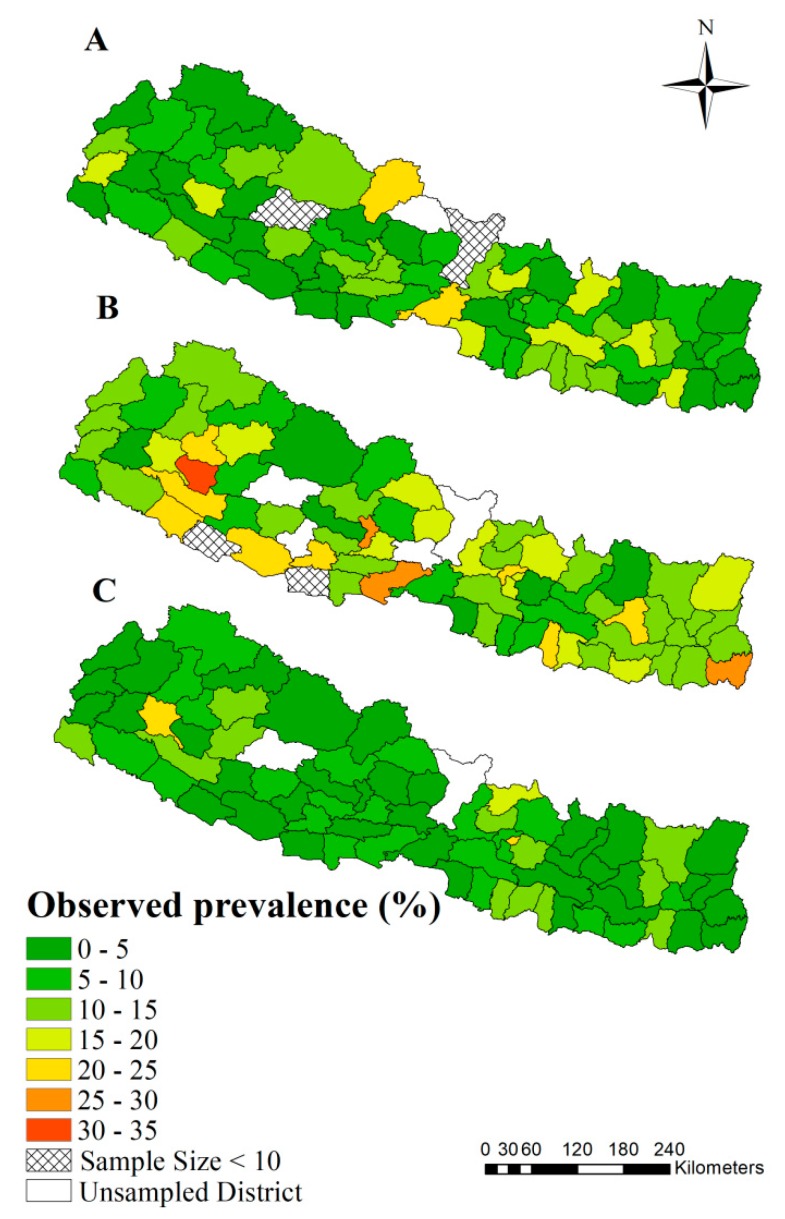
Observed prevalence of diarrhea at district-level among children under five years old in Nepal. (**A**)–(**C**) indicate observed prevalence in the survey years 2006, 2011 and 2016, respectively.

A total of 5783, 5306 and 5038 households were sampled from 74, 71 and 73 districts in the survey year 2006, 2011 and 2016 in Nepal, with 5416, 5028 and 4827 total effective responders after excluding non-responders and “don’t know” responders, respectively. Around 48.2% of survey children were girls, while 51.8% were boys. The observed prevalence, adjusted by survey weights according to the DHS guideline [42], is shown in the Table 2 at a regional level and Figure 2 at the district level. 95% credible intervals for the observed prevalence in each district are listed in Supplement Materials Table S1. Results were consistent with the prevalence reported in the DHS reports.

The observed prevalence of diarrhea was generally decreased in 2016 and shows less heterogeneity between districts, while it varied among districts in 2006 and 2011, with a number of districts show high prevalence (>25%). The districts with very high observed prevalence (>25%) included Chitawan and Mustang in 2006 and Jhapa, Nawalparasi and Parbat Dailekh, Parbat, and Jhapa in 2011. Although there was an overall trend of risk decrease, prevalence in several districts presented an increased trend, such as Rasuwa (11.1%, 12.1% and 18.9% in 2006, 2011 and 2016 respectively) in Central Region and Achham (6.9%, 18.2% and 20.5% in 2006, 2011 and 2016 respectively) in the Far-western Region of Nepal. However, after considering 95% confident intervals, this change seemed not significant (Supplement Materials Table S1).

### 3.2. Single-Factor Analysis

Single-factor analysis showed that proportion of living with mother, proportion of households with improved drinking water sources, proportion of households with improved sanitation, proportion of households using solid fuel, proportion of urban type of residence, seasonality, children’s age, mother’s age when giving birth and mother’s education year were significant different between children under five years old who were reported with diarrhea and those without in two weeks preceding the survey (Table 3). There was no significant statistical difference in environmental and climatic factors between the two groups.

### 3.3. Spatial-Temporal Analysis

Spatial autocorrelation tests (Table 4) showed that there was no significant autocorrelation of diarrhea prevalence at district level. Thus, we assumed unstructured spatial random effects of districts in the final spatial-temporal model. In addition, the model with AR1 structure for the temporal matrix resulted the estimated coefficient ρ within 95% Bayesian credible interval (BCI)-0.627–0.176 (including zero), which indicates the diarrhea risk was not significantly correlated between survey years after adjusting influencing factors, thus the unstructured time-specific random effects were adopted for our final model.

The final Bayesian spatial-temporal model identified that mother’s education year, age, gender of children and seasonality were important influencing factors for diarrhea risk of children under five years old in Nepal (Table 5). The risk decreased with the mother’s education year; children among 12–24 months were with highest risk of diarrhea; boys had higher risk than girls; autumn and winter had lower risk. Although sanitation didn’t show significance in our model within 95% BCI, the posterior probability of the corresponding coefficient larger than zero was only 6.8%, indicating there was a high chance (93.2%) that improved sanitation was a protective factor.

**Figure 3:**
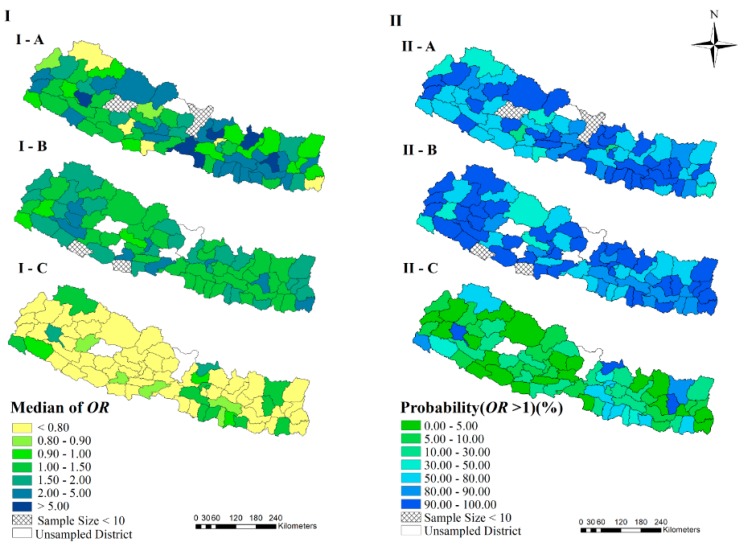
The estimated relative risk (*OR*) of districts, compared with the country-level risk in 2016. (I-A), (I-B) and (I-C) indicate the median of posterior distribution of *OR* in the survey years 2006, 2011 and 2016, respectively. (II-A), (II-B) and (II-C) represent the probability of *OR* larger than one in the survey years 2006, 2011 and 2016, respectively.

Figure 3 showed the relative diarrhea risk in each district ($OR_{jt}$), compared with the estimated country-level risk in 2016. Most of the districts in 2006 and 2011 show higher risk with median of $OR_{jt}>1$, many of which had the probability of $OR_{jt}>1$ higher than 90%. In 2016, there were a large number of districts with median of $OR_{jt}<1$, many of which had the probability of $OR_{jt}>1$ less than 10%. The median of $OR_{jt}$ in Bhojpur, Morang and Rasuwa were higher than 1.2 in all the three survey years. The median of $OR_{jt}$ in Achham and Rasuwa in 2016 were higher than 1.5.

**Figure 4:**
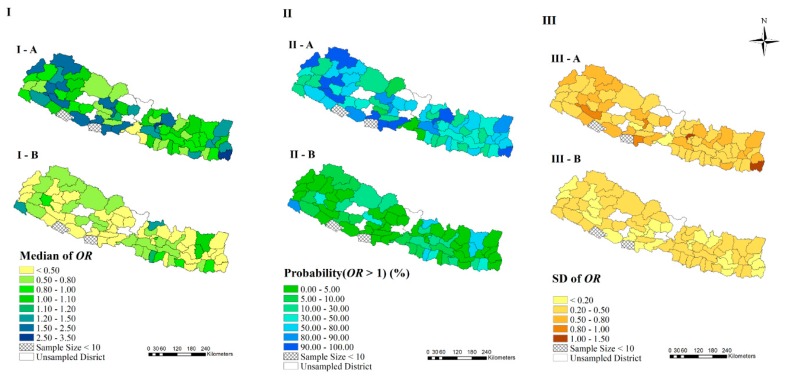
The temporal changes of risk across survey years. (I-A) and (I-B) present the median of posterior distribution of *OR* during the years 2011–2006 and 2016–2011, respectively. (II-A) and (II-B) present the probability of *OR* larger than one for 2011–2006 and 2016–2011, respectively. (III-A) and (III-B) present the *SD* of posterior distribution of *OR* for 2011–2006 and 2016–2011, respectively.

Figure 4 showed the temporal changes of district-risk across survey years ($OR_{j,t_{u-v}}$). There were 32 of 68 districts with decrease of risk, remaining 36 with increase of risk from 2006 to 2011, according to the median of $OR_{j,2011-2006}$; while most districts had a decreased risk from 2011 to 2016, with a few exceptions. Kanchanpur had a chance of increased risk more than 80% from 2011 to 2016 and Bhojpur, Rasuwa, Rautahat and Sankhuwasabha had a chance more than 50%, and among them, the $OR_{j,2016-2011}$ in Kanchanpur and Rusuwa was higher than 1.2 (Figure 4II). Achham showed an increase of risk from 2011 to 2006, with no obvious decrease from 2011 to 2016, based on median of $OR_{j,2011-2006}$ and $OR_{j,2016-2011}$. The *SD*s in Figure 4III, reflecting the uncertainty for *OR*s of temporal change, were less than 0.5 in most districts during the years 2016–2011.

Results for the spatial-temporal random effect term $\delta_{it}$ are shown in Supplementary Materials Figures S1 and S2. In addition, we also did the Moran’s ***I*** test for the median of the posterior estimation of $\delta_{jt}$, which showed no significant spatial autocorrelation either (Supplementary Materials Table S2). For all variance the model could explain, 55.3% of which was explained by covariates and the other 44.7% by the random effects. Proportions of variance explained by covariates in the year 2006, 2011 and 2016 were estimated to 64.0%, 85.9% and 27.3%, respectively, based on separate models by survey year.

## 4. Discussion

In this study, we analyzed the spatial and temporal patterns and explored the important influencing factors of diarrhea among children under five years old in Nepal, with application of the Bayesian spatial-temporal modeling framework. Both the observed prevalence and the estimated relative risk showed a decrease of diarrhea risk from 2006 to 2016 in most districts of Nepal. There were no obvious risk clusters and each district tended to have its own spatial-temporal pattern. Mother’s education year, age, gender and season were important influencing factors of diarrhea in children under five years old in Nepal.

The important influencing factors identified in our final Bayesian spatial-temporal model were consistent with previous studies [43,44]. Our study found that the diarrhea risk decreased with mother’s education year, which was consistent with previous findings [45]. Women’s education may enhance maternal knowledge, attitude and practices during childhood diarrhea [46]. We found that boys had higher risk of having diarrhea than girls, similar as results in studies done in Brazil and Sudan, probably due to greater exposure of boys than girls in unsanitary conditions [47,48]. The diarrhea risk was found first increased and then decreased with age and children in 12–24 months old had the highest risk, corresponding with results from other studies [20,49,50], which might due to combined effects of active immunity, children’s activities and behaviors in different ages [51]. In autumn and winter, there was lower risk of diarrhea compared to spring and summer, possibly due to the dry weather in these two seasons and bacterial pathogens usually peak during hotter and rainier times [22]. There was a high chance (93.2%) that improved sanitation was a protective factor, which is not contradictory with previous studies [52], suggesting improvement of sanitation should not be overlooked for diarrhea control. However, we didn’t find improved drinking water sources as an important influencing factor which was inconsistent with the study in Indonesia [53], probably because of the already high coverage of improved water source in Nepal. In 2016, 95% of households in Nepal could access to improved drinking water sources, but quality of drinking water remained low [54]. Improvement of water supply sources does not necessarily mean accessing to clean drinking water, thus, more sensitive indicators of drinking water, such as drinking water quality, may better reveal the correlation between drinking water and diarrhea. Although there were studies proved that some climate factors were associate with child diarrhea [55,56], in our study we didn’t find any explored environmental/climatic factors significantly different between children reporting having diarrhea and those without in single-factor analysis. On one hand, the environmental factors we explored might not be important influencing factors in Nepal. On the other hand, the relationship of environmental factors and diarrhea might be detected more sensitively if the disease data could be assigned to the environmental data at a higher temporal resolution [22,57], which was not available in the current study.

The overall decrease of diarrhea risk in Nepal was encouraging, probably attributing to the development of politics, launching of national policies and strategies, implementation of control programmes and improvement of sanitation and drinking water sources. Nepal has made significant progress in poverty reduction and human development [58]. With political development, Nepal’s Constitution enshrines the right to healthy living and access to health services as a fundamental human right [59]. The launch of the National Health Policy (2014) and Nepal Health Sector Strategy (2015–2020), putting the Universal Health Coverage at the center, guide the overall health plans for Nepal [60]. The nationwide implementation of community-based programmes, such as the Community Based Integrated Management of Childhood Illness (CB-IMCI), created an enabling environment for better control and treatment of children diarrhea [61]. There were 62% and 95% of households in Nepal using improved sanitation facilities and improved water sources in 2016, respectively [62]. Such improvements from the Water, Sanitation and Hygiene (WASH) programme in Nepal may contribute to the overall decrease of diarrhea risk [63,64].

However, the diarrhea risk was still high or with increased trend in several districts, such as Rasuwa in the Central and Achham in the Far-western region, which should be taken priority when interventions are planning. We compared risk factors between 2011 and 2016 for the above districts and found no obvious regularity. As $\delta_{jt}$ in these districts also showed an increasing trend except Sankhuwasabha, indicating there may be other factors unknown or unobtainable influencing the diarrhea risk. These factors might be district-specific and finding them out are important, particularly for the targeted intervention. Our results urge the governments of these districts paying more attention to the potential reasons for the increasing trend of diarrhea and more efficient control methods should be undertaken.

Even though we considered a large number of potential influencing factors on diarrhea risk, there may be other factors unobserved or unknown, which are heterogeneous in space and time. We introduced spatial-temporal random effects in the Bayesian regression models for taking into account the potential influences of these factors on the disease. Indeed, they accounted for 44.7% of variances the model could explain. A full model was run with spatially and temporally structured effects interacting, results of which (Supplementary Materials Table S3) were similar as that of the model with unstructured spatial-temporal random effects. Thus, we tended to select the simpler one as the final model. Our analysis indicated no obvious spatial-temporal clusters among districts, suggesting district-specific control plans should be considered for more effective control of diarrhea in children under five years old, particularly for those priority districts.

Diarrheal pathogens are usually transmitted through water, food and personal contact with the carrier or contaminated surfaces [65]. With the combination of our findings and previous studies, improvements in sanitation facilities, hygienic food preparation, hand hygiene practice, water quality, quantity and ease of access to water are recommended as preventive measures for the control of diarrhea [43,65,66]. Such measures should be emphasized in intervention programmes, particularly for those priority areas. The functional status of water schemes and the quality of water remains poor in 71% of all water sources in Nepal [67]. In addition, 38% of households were still with unimproved sanitation facility and 20% of government schools were lack of improved water and sanitation facilities in 2016 [62,68], which urges the government to make more efforts on the improvements. Boys and children in age 12–24 months were high risk groups of diarrhea and special attentions should be paid on prevention. However, girls and children in other age groups should not be ignored, and equal care seeking and access to treatment should be made in all groups when diarrhea occurs [69]. In addition, to reduce the prevalence of infant diarrhea, standard breast-feeding and supplementary food should be given, and infant health care should be strengthened [70,71]. Furthermore, improvement of women’s education is critically important, and during spring and summer publicity about hygiene needs to be more strengthened.

Some limitations may exist in our study. The outcome variables were reported by mothers interviewed with the NDHS Women’s Questionnaire and we assumed consistent diagnostic criteria for each mother, which might lead to reporting bias. In addition, without laboratory diagnosis, the types of pathogens could not be differentiated. Different pathogens may have their specific kinds of influencing factors [72,73,74,75], which we were not able to explore in this study. Furthermore, we analyzed the spatial pattern at district level instead of at a higher spatial resolution. DHS did provide geo-referenced data at cluster level, however, the number of mothers interviewed at many survey locations (i.e., clusters) was small (96.4% of survey clusters with sample size less than 30). It’s difficult to have reliable prediction ability for high-resolution risk estimates, by using point-referenced geostatistical methods [25]. Larger number of responders should be included at cluster level in order to make reliable high-resolution maps of diarrhea risk in Nepal. Other public data such as Multiple Indicator Cluster Surveys (MICS) also include information regarding to children diarrhea. However, the open-access data provided by MICS was on sub-regional level instead of district-level [76]. Under the consideration of data consistency and the geographic unit of our spatial-temporal analysis, we only used the DHS data in this study.

## 5. Conclusions

Bayesian spatial-temporal modeling identified age, gender, seasonality and mother’s education year as the important influencing factors on diarrhea among children under five years of age in Nepal, which provides important insights for control and intervention of children diarrhea in the country. Even though a general decreased trend of diarrhea risk, a few districts showed an increased trend. Special attention should be paid to high risk groups of children and high risk seasons, as well as districts with high risk or increased trend of risk (e.g., Ahham and Rusuwa). Effective actions should be implemented to improve sanitation and women’s education level. As each district tended to have its own spatial-temporal pattern, district-specific control planning is recommended for local governments for effective control of children diarrhea in Nepal.

## Figures and Tables

**Table 1:** Summary of environmental and climatic data sources *.

Data Type	Source	Period	Temporal Resolution	Spatial Resolution
NDVI ^a^	MODIS/Terra ^b^	2006, 2011 and 2016	16 days	1 km
LST ^c^ in the daytime and at night	MODIS/Terra ^b^	2006, 2011 and 2016	8 days	1 km
Elevation	WorldClim ^d^	2000	-	1 km
Moisture	Atlas of the Biosphere ^e^	1950–2000	-	50 km
Water bodies	SWBD ^f^	2000	-	30 m

* Data were extracted in September 2018. ^a^ Normalized difference vegetation index. ^b^ Moderate Resolution Imaging Spectroradiometer (MODIS)/Terra, available at: http://modis.gsfc.nasa.gov/. ^c^ Land surface temperature (LST) day and night. ^d^ WorldClim, available at: http://www.worldclim.org/current. ^e^ Biosphere, available at: https://nelson.wisc.edu/sage/data-and-models/atlas/index.php. ^f^ Shuttle Radar Topography Mission Water Body Data (SWBD), available at: http://gis.ess.washington.edu/data/vector/worldshore/index.html.

**Table 2:** Observed diarrhea prevalence among children under 5 years old in development regions of Nepal, categorized by survey years.

Development Regions	2006		2011		2016	
	No. Respondents	Prevalence (%)	No. Respondents	Prevalence (%)	No. Respondents	Prevalence (%)
Eastern	1217	11.83	1148	11.66	902	6.33
Central	1342	12.30	1066	15.02	1264	9.67
Western	1281	12.87	1159	15.63	923	5.39
Mid-western	778	9.32	914	14.37	1078	8.49
Far-western	798	12.07	741	10.94	660	6.22
Total	5416	11.99	5028	13.92	4827	7.67

**Table 3:** Comparison of potential risk factors between children under five years old with and without diarrhea.

Factors		with Diarrhea (n=1674)	without Diarrhea (n=13,597)	*p*-Value
Age ^c^	Age (<12 month)	457 (27.30%)	2496 (18.36%)	<0.001 ^d,^*
	Age (12–24 month)	538 (32.14%)	2493 (18.33%)	
	Age (24–59 month)	679 (40.56%)	8608 (63.31%)	
Gender ^c^	Boy	943 (56.33%)	6960 (51.18%)	0.053 ^c^
	Girl	731 (43.67%)	6637 (48.82%)	
Live with mother ^c^	Yes	1672 (99.88%)	13,521 (99.44%)	0.028 ^c,^*
	No	2 (0.12%)	76 (0.56%)	
Mother’s education year ^b^		0 (7)	2 (8)	0.001 ^d,^*
Mother’s age when gave birth ^b^		25 (7)	26 (7)	0.032 ^d,^*
Number of children under five years old ^b^		2 (1)	2 (1)	0.695 ^d^
Type of place of residence ^c^	Urban	460 (27.5%)	4577 (33.7%)	0.004 ^c,^*
	Rural	1214 (72.5%)	9020 (66.3%)	
Residing status ^c^	legal	1569 (93.73)	12,789 (94.06)	0.629 ^c^
	Illegal	105 (6.27)	808 (5.94)	
Wealth index ^a,b^		−52,160 (96,609)	−47,194 (106,529)	0.078 ^d^
Water source ^c^	Improved	1334 (79.69%)	11,109 (81.70%)	0.047 ^c,^*
	Unimproved	235 (14.04%)	1680 (12.36%)	
Sanitation ^c^	Improved	676 (40.38%)	6693 (49.22%)	<0.001 ^c,^*
	Unimproved	893 (53.35%)	6095 (44.83%)	
Fuel ^c^	Solid	1362 (81.36%)	10,756 (79.11%)	0.006 ^c,^*
	Nonsolid	207 (12.37%)	2032 (14.94%)	
Season ^c^	Spring (Feb–Apr)	739 (44.15%)	4792 (35.24%)	<0.001 ^c,^*
	Summer (Mar–Jul)	683 (40.80%)	5086 (37.41%)	
	Autumn (Aug–Oct)	108 (6.45%)	1745 (12.83%)	
	Winter (Nov–Jan)	144 (8.60%)	1974 (14.52%)	
Elevation ^b^		694.52 (1283.44)	665.68 (1230.64)	0.439 ^d^
NDVI ^b^		0.56 (0.11)	0.56 (0.11)	0.394 ^d^
Moisture ^b^		76.47 (39.30)	81.66 (39.30)	1.000 ^d^
LST_day (°C) ^b^		25.35 (4.83)	25.49 (4.55)	0.534 ^d^
LST_night (°C) ^b^		16.17 (5.97)	16.36 (5.77)	0.194 ^d^
Distance to the nearest water bodies ^b^		0.02 (0.02)	0.02 (0.02)	0.288 ^d^

^a^ The index representing family wealth, combined from household assets collected from DHS surveys [28]; ^b^ presenting as median (interquartile range); ^c^ $\chi^{2}$ test was used; ^d^ Wilcoxon rank-sum test was used; * significant difference.

**Table 4:** Spatial autocorrelation analysis of diarrhea prevalence in children under five years old in Nepal, categorized by the survey year.

Year	Moran’s *I*	Expected Index	Variance	*z*-Score	*p*-Value
2006	0.03	−0.01	<0.01	0.64	0.53
2011	0.02	−0.01	<0.01	0.56	0.58
2016	0.04	−0.01	<0.01	1.01	0.31

**Table 5:** Posterior summaries of the final Bayesian spatial-temporal model parameters.

Covariates	Median (95% BCI)	*OR* (95% BCI)	Prob (%) ^$^
Gender (Girl) ^#^	0.22 (0.12, 0.33) *	1.24 (1.13, 1.39)	>99.99
Age of child (<12 month) ^#^			
12–24 month	0.18 (0.04, 0.32) *	1.20 (1.04, 1.38)	99.80
>24 month	−0.86 (−0.99, −0.73) *	0.42 (0.37, 0.48)	<0.01
Mother’ education year	−0.07 (−0.14, −0.0003) *	0.93 (0.87,0.997)	2.80
Mother’s age when gave birth	−0.03 (−0.09, 0.03)	0.97 (0.91, 1.03)	15.00
Live with mother	1.07 (−0.11, 2.72)	2.91 (0.90, 15.18)	93.6
Type of place of residence (Rural) ^#^	−0.04 (−0.18, 0.10)	0.999 (0.84, 1.15)	32.60
Wealth index	−0.01 (−0.10, 0.08)	0.99 (0.90, 1.08)	40.20
Water source (Unimproved) ^#^	−0.0001 (−0.14, 0.14)	1.0001 (0.81, 1.13)	50.80
Sanitation (Unimproved) ^#^	−0.10 (−0.24, 0.04)	0.79 (0.70, 0.91)	6.80
Fuel (Nonsolid) ^#^	−0.04 (−0.26, 0.18)	0.90 (0.79, 1.04)	36.80
Season (Spring) ^#^			
Summer	−0.14 (−0.29, 0.006)	0.87 (0.75, 1.006)	3.00
Autumn	−0.85 (−1.12, −0.59) *	0.43 (0.33, 0.55)	<0.01
Winter	−0.68 (−0.92, −0.45) *	0.51 (0.40, 0.64)	<0.01
Spatial-temporal variance ($\sigma^{2}$)	7.36 (4.91, 11.70)	-	-

^#^ Baseline is presented in brackets; * significant effect based on 95% Bayesian credible interval; ^$^ posterior probability of the coefficient larger than zero.

## Supplementary Materials

The following are available online at https://www.mdpi.com/1660-4601/17/6/2140/s1, Figure S1: Posterior median (I) and *SD* (II) of the term $\delta_{it}$ for spatial-temporal random effects in Nepal, stratified by the survey years, Figure S2: The temporal change of $\delta_{it}$. (I-A) and (I-B) present the median of posterior distribution of OR between the survey year 2011–2006 and 2016–2011, respectively. (II-A) and (II-B) present the probability of OR>1 for the survey year 2011–2006 and 2016–2011, respectively, Table S1: Prevalence and 95% confidence intervals of observed prevalence in each district by the survey year, Table S2: Spatial autocorrelation analysis of the median of posterior distribution of $\delta_{jt}$, categorized by the survey year, Table S3: Posterior summaries of the final Bayesian spatial-temporal model parameters of full model.

## References

[1] UNICEF, WWBU Levels and Trends in Child Mortality Report 2019. https://www.unicef.org/reports/levels-and-trends-child-mortality-report-2019.

[2] UN News on Millennium Development Goals. https://www.un.org/millenniumgoals.

[3] UN About the Sustainable Development Goals. https://www.un.org/sustainabledevelopment/sustainable-development-goals/.

[4] Kotloff K.L., Platts-Mills J.A., Nasrin D., Roose A., Blackwelder W.C., Levine M.M. (2017). Global burden of diarrheal diseases among children in developing countries: Incidence, etiology, and insights from new molecular diagnostic techniques. Vaccine.

[5] Alebel A., Tesema C., Temesgen B., Gebrie A., Petrucka P., Kibret G.D. (2018). Prevalence and determinants of diarrhea among under-five children in Ethiopia: A systematic review and meta-analysis. PLoS ONE.

[6] WHO Causes of Child Mortality. https://www.who.int/gho/child_health/mortality/causes/en/.

[7] Walker C.L.F., Rudan I., Liu L., Nair H., Theodoratou E., Bhutta Z.A., O’Brien K.L., Campbell H., Black R.E. (2013). Global burden of childhood pneumonia and diarrhoea. Lancet.

[8] Victora C.G.P.D., Requejo J.H.P., Barros A.J.D.P., Berman P.P., Bhutta Z.P., Boerma T.M., Chopra M.M., de Francisco A.M., Daelmans B.M., Hazel E.M. (2015). Countdown to 2015: A decade of tracking progress for maternal, newborn, and child survival. Lancet.

[9] Nepal Statistics. https://www.who.int/countries/npl/en/.

[10] United States of America Statistics Not the Publisher. https://www.who.int/countries/usa/en/.

[11] WHO Proportion of Deaths by Country. Diarrhoeal Diseases. http://apps.who.int/gho/data/view.main.ghe3002015-CH3?lang=en.

[12] Akram R., Sultana M., Ali N., Sheikh N., Sarker A.R. (2018). Prevalence and Determinants of Stunting Among Preschool Children and Its Urban-Rural Disparities in Bangladesh. Food Nutr. Bull..

[13] Guerrant R.L., Schorling J.B., McAuliffe J.F., de Souza M.A. (1992). Diarrhea as a cause and an effect of malnutrition: Diarrhea prevents catch-up growth and malnutrition increases diarrhea frequency and duration. Am. J. Trop. Med. Hyg..

[14] Ministry of Health and Population Nepal, NEII (2016). Nepal Demographic Health Survey 2016.

[15] Publisher Number of Deaths by Country Diarrhoeal Diseases. http://apps.who.int/gho/data/view.main.ghe1002015-CH3?lang=en.

[16] Acharya D., Singh J.K., Adhikari M., Gautam S., Pandey P., Dayal V. (2018). Association of water handling and child feeding practice with childhood diarrhoea in rural community of Southern Nepal. J. Infect Public Health.

[17] Hasan M.M., Richardson A. (2017). How sustainable household environment and knowledge of healthy practices relate to childhood morbidity in South Asia: Analysis of survey data from Bangladesh, Nepal and Pakistan. BMJ Open.

[18] Pruss-Ustun A., Bartram J., Clasen T., Colford J.J., Cumming O., Curtis V., Bonjour S., Dangour A.D., De France J., Fewtrell L. (2014). Burden of disease from inadequate water, sanitation and hygiene in low- and middle-income settings: A retrospective analysis of data from 145 countries. Trop. Med. Int. Health.

[19] Chang A.Y., Riumallo-Herl C., Salomon J.A., Resch S.C., Brenzel L., Verguet S. (2018). Estimating the distribution of morbidity and mortality of childhood diarrhea, measles, and pneumonia by wealth group in low- and middle-income countries. BMC Med..

[20] Strand T.A., Sharma P.R., Gjessing H.K., Ulak M., Chandyo R.K., Adhikari R.K., Sommerfelt H. (2012). Risk factors for extended duration of acute diarrhea in young children. PLoS ONE.

[21] Budhathoki S.S., Bhattachan M., Yadav A.K., Upadhyaya P., Pokharel P.K. (2016). Eco-social and behavioural determinants of diarrhoea in under-five children of Nepal: A framework analysis of the existing literature. Trop. Med. Int. Health.

[22] Chao D.L., Roose A., Roh M., Kotloff K.L., Proctor J.L. (2019). The seasonality of diarrheal pathogens: A retrospective study of seven sites over three years. PLoS Negl. Trop. Dis..

[23] Ikeda T., Kapwata T., Behera S.K., Minakawa N., Hashizume M., Sweijd N., Mathee A., Wright C.Y. (2019). Climatic Factors in Relation to Diarrhoea Hospital Admissions in Rural Limpopo, South Africa. Atmosphere.

[24] Azage M., Kumie A., Worku A.C., Bagtzoglou A., Anagnostou E. (2017). Effect of climatic variability on childhood diarrhea and its high-risk periods in northwestern parts of Ethiopia. PLoS ONE.

[25] Banerjee S., Carlin B.P., Gelfand A.E. (2014). Hierarchical Modeling and Analysis for Spatial Data.

[26] Lawson A.B. (2013). Bayesian Disease Mapping: Hierarchical Modeling in Spatial Epidemiology.

[27] Osei F.B., Stein A. (2019). Bayesian Random Effect Modeling for analyzing spatial clustering of differential time trends of diarrhea incidences. Sci. Rep..

[28] Osei F.B., Stein A. (2017). Diarrhea Morbidities in Small Areas: Accounting for Non-Stationarity in Sociodemographic Impacts using Bayesian Spatially Varying Coefficient Modelling. Sci. Rep..

[29] Anwar M.Y., Warren J.L., Pitzer V.E. (2019). Diarrhea Patterns and Climate: A Spatiotemporal Bayesian Hierarchical Analysis of Diarrheal Disease in Afghanistan. Am. J. Trop. Med. Hyg..

[30] WBG Nepal Income Level. https://data.worldbank.org/country/nepal?view=chart.

[31] Spatial Data Download Country: Nepal. https://www.diva-gis.org/gdata.

[32] DHS The Demographic and Health Surveys (DHS) Program. https://dhsprogram.com/.

[33] Ministry of Health and Population Nepal, NEII (2006). Nepal Demographic Health Survey, 2006.

[34] Ministry of Health and Population Nepal, NEII (2011). Nepal Demographic Health Survey, 2011.

[35] Shrestha S., Haramoto E., Malla R., Nishida K. (2015). Risk of diarrhoea from shallow groundwater contaminated with enteropathogens in the Kathmandu Valley, Nepal. J. Water Health.

[36] Climate of Nepal. http://www.anatravels.com/nepal-climate.php.

[37] Pfeiffer D., Robinson T.P., Stevenson M., Stevens K.B., Rogers D.J., Clements A.C. (2008). Spatial Analysis in Epidemiology.

[38] Moran P.A.P. (1950). Notes on continuous stochastic phenomena. Biometrika.

[39] Knorr-Held L. (2000). Bayesian modelling of inseparable space-time variation in disease risk. Stat. Med..

[40] Blangiardo M., Cameletti M. (2015). Spatial and Spatio-Temporal Bayesian Models with R-INLA.

[41] Rue H., Martino S., Chopin N. (2009). Approximate Bayesian inference for latent Gaussian models by using integrated nested Laplace approximations. J. R. Stat. Soc. Ser. B-Stat. Methodol..

[42] Guide to DHS Statistics. https://www.dhsprogram.com/pubs/pdf/DHSG1/Guide_to_DHS_Statistics_29Oct2012_DHSG1.pdf.

[43] Cairncross S., Hunt C., Boisson S., Bostoen K., Curtis V., Fung I.C.H., Schmidt W. (2010). Water, sanitation and hygiene for the prevention of diarrhoea. Int. J. Epidemiol..

[44] Takele K., Zewotir T., Ndanguza D. (2019). Risk factors of morbidity among children under age five in Ethiopia. BMC Public Health.

[45] Asfaha K.F., Tesfamichael F.A., Fisseha G.K., Misgina K.H., Weldu M.G., Welehaweria N.B., Gebregiorgis Y.S. (2018). Determinants of childhood diarrhea in Medebay Zana District, Northwest Tigray, Ethiopia: A community based unmatched case-control study. BMC Pediatr..

[46] Dhingra D., Dabas A., Anand T., Pinnamaneni R. (2018). Maternal knowledge, attitude and practices during childhood diarrhoea. Trop. Doct..

[47] Melo M.C., Taddei J.A., Diniz-Santos D.R., Vieira C., Carneiro N.B., Melo R.F., Silva L.R. (2008). Incidence of diarrhea in children living in urban slums in Salvador, Brazil. Braz. J. Infect. Dis..

[48] Siziya S., Muula A.S., Rudatsikira E. (2013). Correlates of diarrhoea among children below the age of 5 years in Sudan. Afr. Health Sci..

[49] Ansari S., Sherchand J.B., Parajuli K., Mishra S.K., Dahal R.K., Shrestha S., Tandukar S., Pokhrel B.M. (2012). Bacterial etiology of acute diarrhea in children under five years of age. J. Nepal Health Res. Counc..

[50] Anand A., Roy N. (2016). Transitioning toward Sustainable Development Goals: The Role of Household Environment in Influencing Child Health in Sub-Saharan Africa and South Asia Using Recent Demographic Health Surveys. Front. Public Health.

[51] Melese B., Paulos W., Astawesegn F.H., Gelgelu T.B. (2019). Prevalence of diarrheal diseases and associated factors among under-five children in Dale District, Sidama zone, Southern Ethiopia: A cross-sectional study. BMC Public Health.

[52] Cha S., Lee J., Seo D., Park B.M., Mansiangi P., Bernard K., Mulakub-Yazho G.J.N., Famasulu H.M. (2017). Effects of improved sanitation on diarrheal reduction for children under five in Idiofa, DR Congo: A cluster randomized trial. Infect. Dis. Poverty.

[53] Otsuka Y., Agestika L., Sintawardani N., Yamauchi T. (2019). Risk Factors for Undernutrition and Diarrhea Prevalence in an Urban Slum in Indonesia: Focus on Water, Sanitation, and Hygiene. Am. J. Trop. Med. Hyg..

[54] Aihara Y., Shrestha S., Kazama F., Nishida K. (2015). Validation of household water insecurity scale in urban Nepal. Water Policy.

[55] Chou W., Wu J., Wang Y., Huang H., Sung F., Chuang C. (2010). Modeling the impact of climate variability on diarrhea-associated diseases in Taiwan (1996–2007). Sci. Total Environ..

[56] Wangdi K., Clements A.C. (2017). Spatial and temporal patterns of diarrhoea in Bhutan 2003–2013. BMC Infect. Dis..

[57] Xu Z., Hu W., Zhang Y., Wang X., Zhou M., Su H., Huang C., Tong S., Guo Q. (2015). Exploration of diarrhoea seasonality and its drivers in China. Sci. Rep..

[58] UNDP Nepal Human Development Report 2014. http://www.hdr.undp.org/sites/default/files/nepal_nhdr_2014-final.pdf.

[59] WHO Nepal—WHO Country Cooperation Strategy: 2018–2022. https://apps.who.int/iris/handle/10665/272476.

[60] MoHP Nepal Health Sector Strategy. https://nepal.unfpa.org/en/publications/nepal-health-sector-strategy-2015-2020.

[61] DoHS, MoHP Annual Report of Department of Health Services 2072/73 (2015/2016). https://phpnepal.org.np/publication/current-issue/recently-released/136-annual-report-of-department-of-health-services-2072-73-2015-2016.

[62] Ministry of Health and Population Nepal, NEII Key Indicators of Nepal Demographic and Health Survey 2016. https://phpnepal.org.np/publication/current-issue/recently-released/120-key-indicators-of-nepal-demographic-and-health-survey-2016.

[63] Aryal K.K., Joshi H.D., Dhimal M., Singh S.P., Dhakal P., Dhimal B., Bhusal C.L. (2012). Environmental burden of diarrhoeal diseases due to unsafe water supply and poor sanitation coverage in Nepal. J. Nepal Health Res. Counc..

[64] Kafle S., Pradhan B. (2018). Situation of Water, Sanitation and Hygiene and Diarrhoeal Diseases After Open Defecation Free Declaration. J. Nepal Health Res. Counc..

[65] Ahs J.W., Tao W., Löfgren J., Forsberg B.C. (2010). Diarrheal Diseases in Low-and Middle-Income Countries: Incidence, Prevention and Management. Open Infect. Dis. J..

[66] Clasen T., Schmidt W., Rabie T., Roberts I., Cairncross S. (2007). Interventions to improve water quality for preventing diarrhoea: Systematic review and meta-analysis. BMJ.

[67] Government of Nepal Country Programme Action Plan 2018–2022. https://www.unicef.org/nepal/media/191/file/CPAP%202018-2022.pdf.

[68] Ministry of Education, Nepal Nepal School Sector Development Plan 2016–2023. https://www.globalpartnership.org/content/nepal-school-sector-development-plan-2016-2023.

[69] Målqvist M., Singh C., Kc A., Medicinska F., Medicinska O.F.V., Institutionen F.K.O.B., Internationell M.O.B.I., Uppsala U. (2017). Care seeking for children with fever/cough or diarrhoea in Nepal: Equity trends over the last 15 years. Scand. J. Public Health.

[70] Gizaw Z., Woldu W., Bitew B.D. (2017). Child feeding practices and diarrheal disease among children less than two years of age of the nomadic people in Hadaleala District, Afar Region, Northeast Ethiopia. Int. Breastfeed J..

[71] Ogbo F.A., Agho K., Ogeleka P., Woolfenden S., Page A., Eastwood J., Global Child Health Research Interest Group (2017). Infant feeding practices and diarrhoea in sub-Saharan African countries with high diarrhoea mortality. PLoS ONE.

[72] Lindsay B., Saha D., Sanogo D., Das S.K., Omore R., Farag T.H., Nasrin D., Li S., Panchalingam S., Levine M.M. (2015). Association Between Shigella Infection and Diarrhea Varies Based on Location and Age of Children. Am. J. Trop. Med. Hyg..

[73] Colombara D.V., Faruque A.S.G., Cowgill K.D., Mayer J.D. (2014). Risk factors for diarrhea hospitalization in Bangladesh, 2000–2008: A case-case study of cholera and shigellosis. BMC Infect. Dis..

[74] Njuguna C., Njeru I., Mgamb E., Langat D., Makokha A., Ongore D., Mathenge E., Kariuki S. (2016). Enteric pathogens and factors associated with acute bloody diarrhoea, Kenya. BMC Infect. Dis..

[75] Berendes D., Leon J., Kirby A., Clennon J., Raj S., Yakubu H., Robb K., Kartikeyan A., Hemavathy P., Gunasekaran A. (2017). Household sanitation is associated with lower risk of bacterial and protozoal enteric infections, but not viral infections and diarrhoea, in a cohort study in a low-income urban neighbourhood in Vellore, India. Trop. Med. Int. Health.

[76] MICS6 TOOLS Study Design. http://mics.unicef.org/tools.

