# Supplementary material for: Diarrhea in Under Five Year-old Children in Nepal: A Spatiotemporal Analysis Based on Demographic and Health Survey Data
Source: Int J Environ Res Public Health. 2020 Mar 23;17(6):2140. doi: 10.3390/ijerph17062140 (PMC7142451; doi:10.3390/ijerph17062140)
Supplement: Supplementary file 1 [file ijerph-17-02140-s001.pdf]

Supplement Materials:

Table S1. Prevalence and 95% confidence intervals of observed prevalence in each district by the survey year.

Region	District	2006		2011		2016	
		Sample size	Prevalence (95%CI)	Sample size	Prevalence (95%CI)	Sample size	Prevalence (95%CI)
Central	Bara	114	10.75 (4.89, 16.60)	139	10.11 (6.19, 17.18)	129	5.28 (2.14, 10.86)
Central	Bhaktapur	21	17.02 (3.05, 36.32)	29	15.61 (3.92, 31.66)	31	21.85 (3.63, 29.84)
Central	Chitawan	145	29.42 (18.63, 33.42)	131	7.91 (4.29, 14.52)	26	4.36 (0.20, 19.07)
Central	Dhading	74	16.07 (7.67, 25.04)	55	17.41 (9.08, 30.91)	22	9.09 (1.09, 29.06)
Central	Dhanusa	45	15.91 (6.48, 29.48)	67	15.03 (7.40, 25.78)	41	4.56 (0.63, 16.47)
Central	Dolakha	110	22.67 (13.76, 29.70)	113	13.72 (7.60, 21.04)	112	3.33 (0.98, 10.00)
Central	Kathmandu	96	1.21 (0.2, 10.02)	84	23.42 (13.22, 31.76)	117	7.90 (3.12, 12.94)
Central	Kavrepalanchok	33	10.14 (1.84, 24.32)	41	3.46 (1.49, 19.94)	38	13.52 (4.41, 28.06)
Central	Lalitpur	110	11.92 (6.46, 19.36)	66	16.67 (8.62, 27.84)	151	6.80 (3.21, 11.83)
Central	Mahottari	154	17.46 (10.24, 22.31)	42	21.43 (10.30, 36.83)	129	10.40 (5.48, 16.68)
Central	Makwanpur	115	5.25 (3.04, 13.24)	103	10.52 (6.17, 19.44)	133	9.58 (5.31, 16.13)
Central	Nuwakot	69	22.15 (13.88, 34.90)	30	10.81 (2.14, 26.52)	49	10.43 (3.39, 22.23)
Central	Parsa	58	22.87 (13.82, 37.17)	38	2.63 (0.07, 13.38)	14	0.00 (0.00, 23.00)
Central	Ramechhap	29	10.50 (2.19, 27.35)	40	7.50 (1.60, 20.37)	33	3.10 (0.07, 15.27)
Central	Rasuwa	89	11.12 (6.34, 21.05)	58	12.07 (4.99, 23.29)	140	18.89 (11.91, 25.23)
Central	Rautahat	55	13.51 (6.50, 26.67)	51	7.84 (2.18, 18.86)	47	10.45 (3.59, 12.08)
Central	Sarlahi	57	15.04 (7.47, 29.88)	48	8.33 (2.31, 19.98)	22	12.03 (2.91, 34.93)
Central	Sindhuli	54	21.43 (9.25, 31.43)	25	5.77 (2.52, 31.21)	21	0 (0.00, 16.00)
Central	Sindhupalchok	88	8.15 (3.23, 15.73)	63	16.15 (9.08, 29.08)	53	8.03 (0.63, 16.47)
Eastern	Bhojpur	101	16.90 (9.35, 24.40)	112	11.47 (6.32, 18.96)	124	12.18 (8.21, 21.02)
Eastern	Dhankuta	35	4.91 (0.70, 19.07)	54	11.11 (4.18, 22.63)	16	6.25 (0.15, 29.49)
Eastern	Ilam	133	8.06 (4.24, 14.36)	54	14.51 (5.39, 24.90)	111	3.72 (0.58, 10.03)
Eastern	Jhapa	93	6.00 (1.77, 12.11)	48	26.16 (16.97, 44.04)	136	3.93 (1.22, 10.01)
Eastern	Khotang	126	20.83 (13.89, 28.75)	74	20.83 (12.90, 32.70)	75	5.00 (1.47, 13.09)
Eastern	Morang	51	8.99 (5.73, 26.25)	106	11.32 (5.99, 18.95)	10	0 (0.00, 31.00)
Eastern	Okhaldhunga	43	19.21 (10.02,36.04)	104	10.23 (5.39, 18.14)	19	0 (0.00, 18.00)
Eastern	Panchthar	55	12.43 (5.27, 24.46)	70	14.29 (7.04, 24.70)	14	0 0.00, 23.00)
Eastern	Sankhuwasabha	112	14.50 (7.01, 20.08)	128	13.30 (8.60, 21.30)	82	10.08 (5.93, 22.71)
Eastern	Saptari	82	6.89 (4.31, 18.38)	42	16.67 (6.98, 31.36)	53	7.63 (2.10, 18.21)
Eastern	Siraha	144	16.42 (9.25, 21.43)	122	11.19 (5.80, 17.51)	118	7.38 (4.13, 15.04)
Eastern	Solukhumbu	37	7.40 (1.67 21.88)	13	0.00 (0.00, 25.00)	49	0.00 (0.00, 8.00)
Eastern	Sunsari	41	21.89 (10.56, 37.63)	43	11.63 (3.89, 25.06)	48	10.43 (3.47, 22.66)
Eastern	Taplejung	96	8.74 (2.32, 13.09)	104	16.72 (7.57, 21.54)	34	1.63 (0.07, 14.85)
Eastern	Terhathum	14	7.14 (0.18, 33.08)	22	13.64 (2.91, 34.90)	17	0.00 (0.00, 20.00)
Eastern	Udayapur	86	12.58 (6.55, 21.74)	136	11.03 (6.30, 17.55)	63	3.11 (3.97, 10.97)

Western	Arghakhanchi	125	9.32 (5.03, 16.17)	87	24.14 (15.60, 34.50)	119	8.22 (4.12, 14.91)
Western	Baglung	41	10.61 (2.73, 23.08)	10	0.00 (0.00, 31.00)	24	3.58 (0.10, 20.53)
Western	Gorkha	2	0/2 *	-	-	-	-
Western	Gulmi	168	16.70 (11.38, 23.19)	157	10.83 (6.46, 16.77)	125	6.97 (2.28, 11.20)
Western	Kapilbastu	47	5.05 (1.38, 17.51)	9	1/9*	27	0.00 (0.00, 13.00)
Western	Kaski	35	7.63 (1.78, 23.04)	36	8.27 (3.10, 26.05)	62	0.00 (0.00, 7.25)
Western	Lamjung	70	11.03 (6.04, 23.01)	57	18.28 (8.72, 29.92)	69	0.85 (0.01,10.01)
Western	Manang	-	-	36	16.67 (6.37, 32.81)	12	8.33 (0.18, 37.66)
Western	Mustang	50	25.59 (11.52, 35.96)	28	8.90 (2.27, 28.26)	39	0.00 (0.00, 9.00)
Western	Myagdi	67	8.40 (1.65, 14.56)	74	12.84 (6.67, 23.46)	70	8.79 (2.36, 15.91)
Western	Nawalparasi	173	12.01 (6.29, 15.94)	127	25.10 (14.50, 29.41)	140	7.14 (3.48, 12.75)
Western	Palpa	91	17.00 (8.68, 24.46)	116	10.34 (5.46, 17.42)	33	1.47 (0.10, 15.28)
Western	Parbat	18	16.67 (3.50, 41.40)	28	28.57 (13.24, 48.67)	49	2.48 (0.06, 10.50)
Western	Rupandehi	74	2.83 (0.85, 11.42)	79	14.73 (8.10, 25.02)	126	8.24 (3.87, 14.12)
Western	Syangja	37	17.95 (7.99, 35.14)	34	17.65 (6.76, 34.52)	47	6.11 (1.35, 17.51)
Western	Tanahu	21	14.29 (3.05, 36.34)	-	-	52	1.86 (0.02, 10.01)
Mid-Western	Banke	44	10.54 (1.44, 18.64)	6	0/6*	20	4.18 (0.12, 24.21)
Mid-Western	Bardiya	26	15.38 (42.85, 34.85)	27	22.22 (8.65, 42.25)	14	7.14 (0.17, 33.09)
Mid-Western	Dailekh	27	22.22 (8.61, 42.27)	22	31.82 (13.89,54.87)	23	0.00 (0.00, 15.00)
Mid-Western	Jajarkot	31	6.45 (0.81, 21.34)	48	8.33 (2.28, 19.99)	53	12.29 (4.27, 23.02)
Mid-Western	Surkhet	39	7.69 (1.59, 20.85)	80	23.75 (14.95, 34.57)	35	11.27 (3.19, 26.72)
Mid-Western	Dolpa	28	17.86 (6.11, 36.88)	45	0.00 (0.00, 8.00)	25	4.54 (0.10, 19.78)
Mid-Western	Humla	122	4.05 (1.86, 10.38)	153	13.78 (9.75, 21.71)	82	9.01 (3.50, 16.75)
Mid-Western	Jumla	76	16.92 (9.43, 27.46)	59	16.95 (8.45, 28.97)	34	11.10 (3.30, 27.45)
Mid-Western	Kalikot	40	14.89 (5.71, 29.85)	76	23.68 (14.67, 34.82)	57	6.60 (1.95, 17.02)
Mid-Western	Mugu	56	3.90 (1.12, 14.87)	29	6.90 (0.85, 28.97)	76	3.81 (0.79, 11.10)
Mid-Western	Dang	21	9.52 (11.78, 30.28)	49	20.44 (10.25, 22.69)	93	0.00 (0.00, 4.95)
Mid-Western	Pyuthan	66	0.00 (0.00, 6.84)	-	-	26	0 (0.00, 13.00)
Mid-Western	Rolpa	79	16.78 (7.15, 23.57)	71	12.94 (5.00, 21.07)	76	7.38 (3.00, 16.37)
Mid-	Rukum	9	0/9*	-	-		-

Western							
Mid-Western	Salyan	13	7.69 (0.19, 35.26)	27	7.41 (0.86, 24.19)	24	0.00 (0.00, 14.00)
Far-Western	Achham	104	6.89 (3.40, 14.58)	153	18.19 (10.30, 22.44)	99	20.54 (11.10, 27.22)
Far-Western	Baitadi	23	17.39 (4.93, 38.78)	108	12.96 (7.29, 20.81)	59	1.94 (0.04, 10.01)
Far-Western	Bajhang	133	10.00 (7.02, 18.78)	117	8.85 (4.78, 16.21)	68	2.40 (0.34, 10.18)
Far-Western	Bajura	97	10.08 (5.05, 18.15)	117	11.40 (7.35, 20.27)	113	8.03 (3.07, 13.46)
Far-Western	Dadeldhura	70	20.17 (11.43, 31.28)	42	11.90 (3.98, 25.65)	41	4.99 (5.80, 16.45)
Far-Western	Darchula	139	6.28 (2.98, 11.93)	119	14.53 (7.91, 20.93)	51	4.36 (0.47, 13.40)
Far-Western	Doti	47	8.51 (23.61, 20.34)	38	2.63 (0.12, 13.38)	91	4.72 (1.20, 10.88)
Far-Western	Kailali	82	11.11 (4.34, 18.31)	123	13.01 (8.29, 21.16)	215	6.90 (4.67, 12.33)
Far-Western	Kanchanpur	244	9.80 (5.71, 13.35)	186	7.12 (3.38, 10.99)	181	10.54 (6.88, 16.56)

*Presented as number of diarrhea/number of responders, due to small number (<10) of responders.

Table S2. Spatial autocorrelation analysis of the median of posterior distribution of δ_{jt}, categorized by the survey year.

Year	Moran's *I*	Expected Index	variance	*z*−score	*p*−value
2006	−0.014451	−0.013699	0.004800	−0.010856	0.991339
2011	−0.011255	−0.014286	0.003478	−0.052144	0.958414
2016	0.070755	−0.013889	0.003162	1.505359	0.132232

Table S3. Posterior summaries of the final Bayesian spatial-temporal model parameters of full model$.

Covariates	Median (95% Bayesian credible interval)	*OR* (95%BCI)
Gender (Girl) #	0.22 (0.12, 0.33) *	1.24 (1.13, 1.39)
Age of child (<12month) #		
12–24 month	0.18 (0.04, 0.32) *	1.20 (1.04, 1.38)
>24 month	−0.86 (−0.99, −0.73) *	0.42 (0.37, 0.48)
Mother' education year	−0.07 (−0.14, −0.003) *	0.93 (0.87,0.997)
Mother's age when gave birth	−0.03 (−0.09, 0.02)	0.97 (0.91, 1.03)
Live with mother	1.08 (−0.11, 2.72)	0.97 (0.91, 1.02)
Type of place of residence (Rural)#	−0.04 (−0.17, 0.10)	0.999 (0.87, 1.15)
Water source (Unimproved)#	0.002 (−0.14, 0.14)	1.002 (0.81, 1.13)
Sanitation (Unimproved) #	−0.10 (−0.24, 0.04)	0.79 (0.70, 0.91)
Fuel (Nonsolid) #	−0.04 (−0.26, 0.18)	0.90 (0.79, 1.04)
Season (Spring)#		
Summer	−0.14 (−0.29, 0.01)	0.87 (0.75, 1.01)
Autumn	−0.85 (−1.11, −0.58)	0.43 (0.33, 0.56)
Winter	−0.66 (−0.90, −0.42)	0.52 (0.41, 0.66)
iid component	7.50 (4.83, 11.97)	-
Spatial component	1361.91 (135.42, 7150.97)	-
ρ	−0.26 (−0.63, 0.17)	-

$The full model assigned $\boldsymbol{R_d}$ through the intrinsic conditional autoregressive (iCAR) specification and t $\boldsymbol{R_t}$ a structure of autoregressive model of order 1 (AR1). #Baseline is presented in bracket; *significant effect based on 95% Bayesian credible interval.

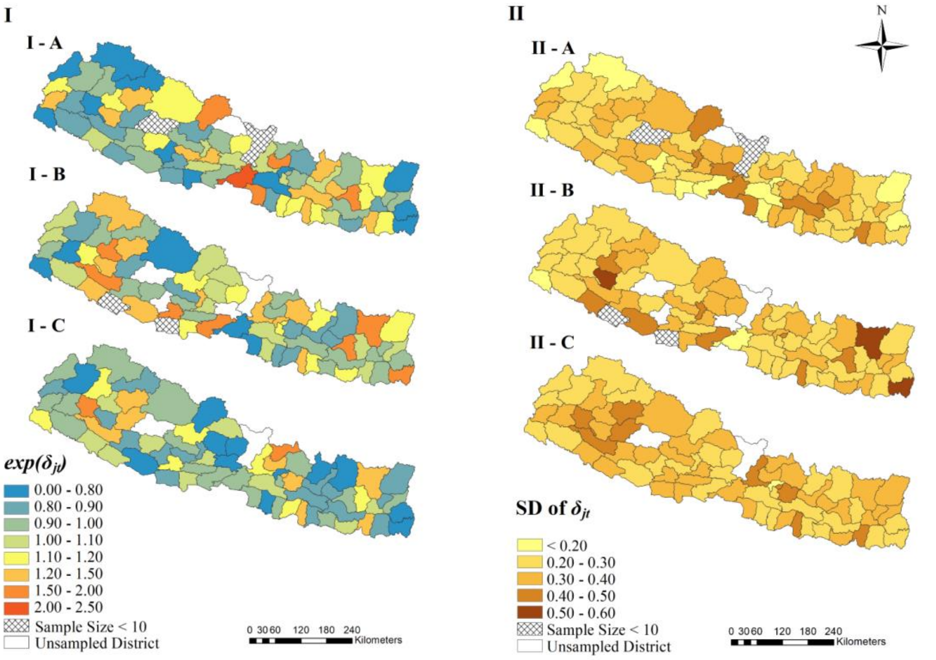

Figure S1. Posterior median (I) and SD (II) of the term δ_{it} for spatial-temporal random effects in Nepal, stratified by the survey years.

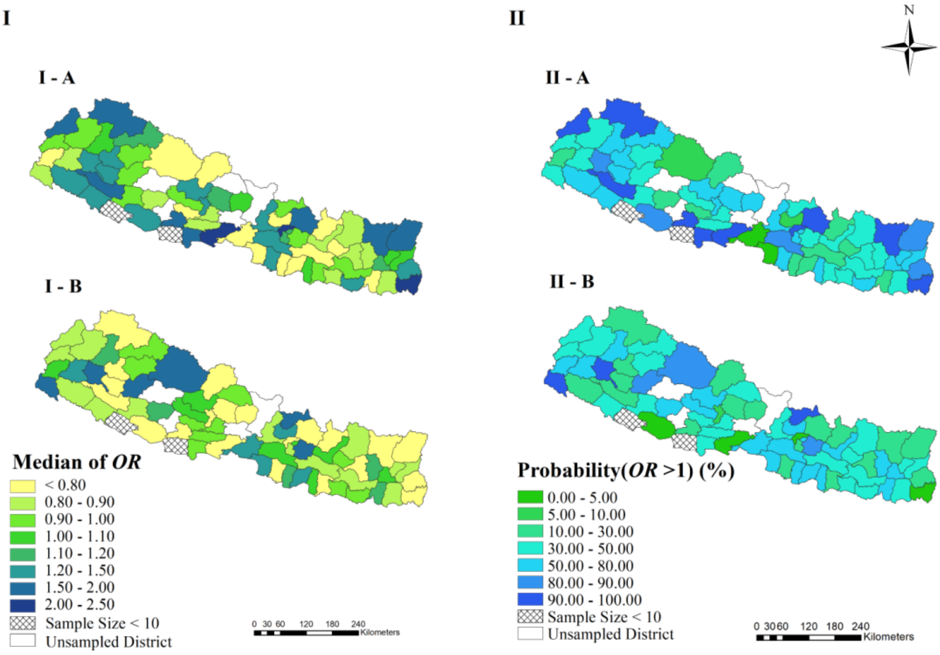

Figure S2. The temporal change of δ_{it}. (I-A) and (I-B) present the median of posterior distribution of OR between the survey year 2011−2006 and 2016−2011, respectively. (II-A) and (II-B) present the probability of $OR > 1$ for the survey year 2011–2006 and 2016−2011, respectively.
